# Index Medicus

**Published:** 1884-01

**Authors:** 


### "INDEX MEDICUS."

It is not dental journals alone that sometimes suffer from a lack of support. The complaint is chronic among medical publications, and it would sometimes seem that the more meritorious they are the more they are allowed to languish. The publisher of that very important work, *Index Medicus,* sends out an appeal to the profession, and an intimation that unless it be better supported its publication must be suspended. Are there not some readers of this journal who will pay ten dollars per year before they will allow such a misfortune ? Will not some society send in a subscription for the benefit of all its members?

